# Veterinary Dental Surgery*Read at the meeting of the Veterinary Association of Manitoba, Canada, on July 22d.

**Published:** 1893-01

**Authors:** J. F. Fisher

**Affiliations:** Brandon, Manitoba, Canada


### VETERINARY DENTAL SURGERY.*

By J. F. Fisher, V.S., Brandon, Manitoba, Canada.

The subject of my paper this evening, viz., "Veterinary Dental Surgery," is one in which the profession of late years has taken deep interest. A knowledge of the derangements and diseases of the teeth is indispensable to the veterinarian, owing to the important relations which these organs bear to the system in general; and although there yet remains a great field for the student to cast light on the mysteries in which certain diseases of the teeth are still more or less involved, and for the ingenious inclined to perfect instruments to facilitate the work of the operator in performing the surgical portion of the treatment, yet during the past few years a great advancement has been made by veterinarians in that branch of our profession, known as Veterinary Dental Surgery.

*Position for Operating.*—The standing position is to be preferred in operating in all practicable cases, for however cautious the surgeon may be, if the lying position is adopted, owing to the manner in which the head is placed while operating—the crest downward and muzzle upward—accidents may arise from diseased teeth and from tooth fragments dropping into the throat and being swallowed, which may result in the production of intestinal calculi and tympanitic affections; also in cleansing the sinuses of the head, the fluid is apt to pass into the larynx, and, finally, the lungs. The speculum should be used as little as possible, as it often produces excoriations of the gums and bars, and sometimes exfoliation of the denuded bone. The mouth should be kept open and all floats, molar cutters and extractors handled by means of the hand and arm in the following manner: If operating on the lower molars, pass three fingers alongside the tongue, pressing it over between molars of opposite side, the other finger and thumb to be used in giving pressure on float and guiding molar cutters and extractors, with arm in interdental space. When operating on upper molars, press thumb against first molar, fingers passing up alongside of cheek letting float run between thumb and fingers with arm in interdental space. When operating on upper incisors, place arm in interdental space, and pass thumb and first finger around incisor teeth, raising upper lip. Handle lower incisors in same way.

Horses from youth to old age should have their mouths occasionally examined to see that the teeth are in right condition; for

* Read at the meeting of the Veterinary Association of Manitoba, Canada, on July 22d.

unless the food is properly masticated, the animal does not receive that nutrition which it requires.

*Diseases of Dentition.*—The effects of dentition on the system generally, have perhaps received too little attention. Experience teaches us, however, that in cough, fever, diarrhœa, dysentery, diseases of the eye, loss of appetite and condition, catarrhal and bronchial inflammations, lymphatic and glandular tumors about the head, cutaneous eruptions and general derangement of the constitution, we will frequently find the producing cause to be some irritation from teething. The symptoms most likely to attract attention would be difficulty of mastication or deglutition, stench of the breath or buccal secretion, and discharge of saliva from the mouth, with occasional slobbering. Upon examining the mouth, the gums may be found to be hot, painful, and stretched from the pressure of the teeth against them, or the unshed crowns of temporary teeth may be found to be entangled with the new teeth, thus causing a source of irritation interfering with the feeding of the animal. The cutting of the canine teeth is most likely to cause trouble, and should be well looked after. Horses at four years old are often subject to a distressing cough, due to the eruption of so many teeth at this age, which causes excessive dental irritation, often extending from the gums of the sixth molar to the fauces and larynx. Lampas also often occurs from the process of growth going on in the teeth, inflaming the gums—which is propagated to the bars, or from some febrile tendency in the constitution. Supernumerary or wolf teeth—the meaning of the term being hurtful or destructive—are sometimes to be met with. They produce irritation of the gums, injury to the eyes, affecting chiefly the mucous membranes and lachrymal glands, causing a weeping from same, through nervous connection, and when they encroach on the maxillary branch of the fifth pair of nerves, they produce symptoms of insanity. The treatment of dentitional diseases consists in careful dieting, with gentle alteratives, lancing the fevered gums, and extracting the obstructing temporary and supernumerary teeth.

*Irregularities of the Teeth* are caused by: (1.) Anomalies in the proper number; the development of the abnormal teeth, situated within or without the rows or in front of the normal range of grinders, is an obstacle to the cheek when on the outside, the tongue when on the inside, and their growth not being counteracted by wear, they lacerate the mucous membrane, interfere with the action of the lower jaw, and the exact apposition and friction of the teeth.

(2.) *Irregularities of the Rows.*—The lower rows of grinders form nearly two straight lines, converging towards the symphysis of the chin, and the upper rows two curves; these, owing to congenital malformation or injuries, may be of such a nature as to interfere with the surfaces of friction, the result being abnormal development of the borders of the crowns on the inside of the lower and the outside of the upper molars. This defect is also often produced by the lateral movement of lower jaw not being effected throughout the limits of the segment of the circle, such being necessary to cause friction, the upper grinders being wider than the lower. Vicious inclinations of the tables of the teeth are occasionally met with in the tables—forming planes so much inclined that they close together like a pair of shears; the teeth in such cases grow to an unnatural length, no friction being produced to cause wear, the half-masticated food in consequence slipping into the pouch of the cheek. Lack of regularity in the length of the rows produces a peculiar deformity in the first upper and last lower molar. The projecting part growing until it reaches the opposite jaw, produces serious consequences if not immediately attended to. When a tooth is deficient or partly deficient, whether from fracture, caries, or arrestation of growth, it is gradually destroyed by the opposite tooth, which grows until it entirely fills the space, and then, if not interfered with, commences its work of destruction on the maxillary bone. Another deformity, not very uncommon, is where the lower teeth wear with greater rapidity than the upper, due to the latter being of greater size and strength; and when the wear extends to the sockets, mastication of hard food is next to impossible. For this defect there is no remedy, but the progress of destruction may be retarded by the use of soft food.

(3.) *Irregularity of Incisor Teeth.*—The development of abnormal teeth and obstructing temporary teeth is to be met with in the incisors as well as the molars. The deformity called parrot-mouth also often occurs, the evil effects of which are met with as soon as the lower incisors become so long as to bruise and otherwise injure the bars.

*Symptoms of Irregularities of the Teeth.*—Difficulty of mastication; the animal excited by hunger takes the food with avidity, but the motion of the lower jaw is often made only at one side, and with a sort of hesitation. The food not being properly masticated will not pass through the narrow pharynx, and the mouthful is again dropped into the manger; the coat soon becomes dry and staring, pulse weak, and the animal sweats on the least exertion, this being due to insufficient nutrition. But when the jaw-bones are contused, the gums inflamed, and the buccal membrane excoriated, the animal loses his appetite, becomes dull and agitated, with febrile disturbance; stringy saliva dribbles from the mouth, which is fœtid, when mixed with pus, the mouth is hot, and the membrane injected; there is swelling of the gum at the point of inflammation and tumefaction of the bone. The diagnosis is precisely determined when the mouth is examined, for then can be perceived the particular signs of each of the alterations that oppose the function of mastication. The examination of the mouth should be made as follows: In examining left side, pass left hand alongside of tongue, pressing it over between molars of opposite side, then cleanse nicely with water. The sight may then be sufficient to detect swollen sockets, vicious inclinations or projections of the tables, fractured, elongated, or deficient teeth, an accumulation of greenish remains of food at affected part, and the existence of a blackish spot or cavity on one of the faces of the tooth, particularly the crown. If its nature cannot be discovered by the eye, it will be necessary to resort to the sense of touch, which is done by feeling with the first finger for the seat of trouble. In testing for carious teeth, keep mouth open with left hand as aforesaid, then take molar cavity cleaner in right hand, pass up alongside of left hand, and lightly tap each tooth, carefully noticing if any pain is evinced. Handle right side in same manner, using right hand in mouth.

*Side and Hard Pullers.*—This defect is due to the bit pressing the cheeks against some of the sharp projections of the molar teeth, thereby lacerating them, and is often to be met with in the deformity of the first upper molar projecting over the lower, the sharp, overlapping point piercing the cheek or tongue, whenever pressure is brought to bear on the bit. It is also sometimes due to the space between the canine teeth being too narrow for the tongue, the bit in consequence not coming in sufficient contact with the the sensitive bars, and the tongue being kept constantly in a state of irritation, by coming in contact with the canine teeth. The remedy for this is to cut off the canine teeeth with bone forceps, remove all sharp molar projections with closed cutter, and round edges with float.

*Caries of the Teeth.*—Caries, dental gangrene or decay, is seldom met with in the incisor teeth ; the grinders are those which almost invariably suffer. It may primarily attack the crown, neck, or root of the tooth, but wherever its primitive seat may be, the blackish seams always extend into the dentine, and thus isolate the plies of enamel. Caries usually attacks the dentine of the crown, between two folds of enamel. It is usually produced by some hard material pressed between the tables, causing fracture of the enamel, and such injury to the dentine that it dies, or has not the power to resist the chemical action of the fluids of the mouth. The decomposition of the dentine and the putrefaction of saliva in the cavity emits an offensive odor. The decay progresses between the folds of enamel, which, notwithstanding its great density, takes on the blackish tint of the dentine, and becomes sufficiently softened to admit of its being cut by a sharp instrument. Carious teeth rarely preserve their form or volume. When the disease penetrates to the root, it becomes hypertrophied, the alveolar dental membrane becomes irritated by the decayed tissue, and deposits osseous matter around the root, until it presents a succession of osseous tubercles, which bar in the tooth and render its extraction difficult. The root, augmented in volume, can no longer be contained in the cavity, the walls of which expand, causing extreme pain in the adjacent parts; the osseous tissue tumefies, and suppuration is established in the interior of the socket; the membrane is partly destroyed, which leaves the bone bare and exposed to the pus and irritating matter which penetrates into the socket by the dental fistula. The bony tissue sphacelates on the borders where its substance is the most compact, and its spongy tissue—which forms the bottom of the cavity—soon becomes the seat of an interstitial suppuration, the swelling sometimes extending throughout the entire maxillary bone. Caries of the roots is rare, and chiefly arises from external injury or a constitutional predisposition. Caries of the neck is much more common than that of the root, and is caused by the food decomposing in the interspaces between the teeth, exciting inflammation in the periodental membrane. The position of the diseased tooth should always be taken into consideration. The two first upper molars are separated from the nasal cavities by a thin bone which is easily eaten through. When caries attacks their roots, the inflammation extends to the membrane lining these cavities, and the perforation of the osseous partition establishes a communication between the mouth and nose which permits the food to pass through the dental fistula into the nasal chambers. The 3rd molar is situated near the maxillary sinus, from which the root is separated by a thin diaphragm; when caries affects its roots, severe pain is often caused by nervous complications; the larger fasciæ of the superior maxillary branch of the 5th, which makes its exit upon the face by the submaxillary foramen, are placed immediately over its root. The 4th, 5th and 6th molars are situated immediately below the maxillary sinuses. These communicate with the sinuses as easily as the first and second do with the nose; but the case is far worse for the horse, there being so little outlet for the pus.

*Symptoms of Caries*—

1st. Fœtor of the mouth and the secretions within it.

2nd. Dribbling of an abundant and stringy saliva from the mouth.

3rd. Existence on one of the faces of the tooth, and principally upon its crown, either of a blackish spot or cavity, according to the extent of the disease.

4th. The extreme pain the animal evinces when the tooth is struck.

5th. Swelling of gums; redness and pain around diseased tooth.

6th. Accumulation of food around the seat of disease, producing a repulsive odor.

*Treatment of Caries.*—The only remedy for caries is the extraction of the tooth, which is performed either with the forceps, or by trephining and punching the tooth out, the method to be preferred depending altogether on the case. After the operation, to stop the hæmorrhage, to modify the state of the membrane, and to guard against caries of the maxillary bone, the alveolus should be cauterized with the actual cautery, or in cases where this cannot be applied, a strong solution of argenti nitras applied with pledgets of tow or lint. The mouth should be cleansed with an acidulated gargle, and the animal fed on gruel and bran-mashes. On the day following the operation, a detergent, such as a chlorinated solution of soda, should be injected into the cavity, and afterwards a pledget of tow, saturated with the same, should be introduced into the socket to prevent anything passing from the mouth to the sinus.—*The Veterinary Journal.*

